# A metacognitive approach to learning and performance in human-AI interaction

**DOI:** 10.1038/s44271-026-00548-y

**Published:** 2026-10-30

**Authors:** Yizhou Fan, Lixiang Yan, Inge Molenaar, Samuel Greiff, Dragan Gašević

**Affiliations:** 1 Graduate School of Education, Peking University, Beijing, PR China; 2 School of Education, Tsinghua University, Beijing, PR China; 3 Behavioural Science Institute, Radboud University, Nijmegen, Netherlands; 4 School of Social Sciences and Technology, Technical University of Munich, Munich, Germany; 5 Centre for International Student Assessment, Technical University of Munich, Munich, Germany; 6 Faculty of Education and School of Computing & Data Science, The University of Hong Kong, Hong Kong, China; 7 Faculty of Information Technology, Monash University, Melbourne, Australia

**Keywords:** Education, Human behaviour

## Abstract

Generative artificial intelligence (GenAI) is rapidly becoming a pervasive technology across education, work, research, and everyday life. Yet growing evidence suggests that fluent, coherent, and seemingly transparent AI assistance can improve task performance while weakening the metacognitive engagement and deeper processing required for durable learning. This tension raises a central question about how does offloading to AI reshape human learning, and under what conditions do performance and learning decouple? In this Perspective, we introduce the Synergetic Notion of Human-AI Skills and Knowledge Construction (SYNC) model. The model offers a metacognitive account of human-AI interaction by conceptualising metacognitive offloading and metacognitive onloading. The SYNC model aims to explain how human-AI interaction unfolds across learning and performance dimensions, clarifying how learners may drift into overreliant AI-supported performance or move toward human-AI synergy. We further identify key pitfalls in ineffective human-AI interaction and outline strategies to address them through metacognitive onloading and support human-AI synergy.

## Introduction

In human history, each major shift in how people produce, communicate and govern across the agrarian, industrial, and information eras has re-specified the mechanisms of human learning and performance^1–9^. Against this long arc, the rise of artificial intelligence (AI), especially generative AI (GenAI) that can produce text, code, images and analysis on demand, marks another inflexion point^10,11^. This inflexion point raises a central question for psychology, education, and technology about how should durable human learning be cultivated so that humans can perform effectively alongside AI technology?

The use of GenAI has already begun to reshape how knowledge and skills are formed, practised, and assessed^12,13^. Large language models (LLMs) can scale personalised tutoring, diversify resources, provide timely formative feedback, and enable conversational, authentic assessments^14–17^. Used well, these capabilities hold the promise to expand access and allow learners to work within their zone of proximal development^18,19^. When GenAI provides high-quality interventions, personalised examples and scaffolded support rather than simply completing tasks for learners, it can enhance humans’ skill development^20–22^. Yet the same technology is imperfect. LLMs hallucinate and inherit biases from the training data^23–26^. The development and use of LLMs raise privacy and fairness concerns^10^, may widen digital divides^27^, and destabilise traditional assessments of human learning by blurring authorship and effort^12,28^.

These ambivalent effects are documented across various learning contexts and motivate a shift from merely using AI tools to deliberately cultivating the human skills necessary to use them effectively^10,29^. Among these human skills, metacognition is especially important for supporting durable learning because it enables learners to plan, monitor, and evaluate their use of GenAI^30–33^. In such contexts, human-AI interactions should therefore be designed to support metacognition through scaffolding^30–32,34^. Two cautions are therefore paramount. First, GenAI can encourage over-reliance and autopilot performance (offloading both cognitive processes and metacognitive control to AI)^35^, which recent research terms as metacognitive laziness^31^. Second, short-term performance gains should not be mistaken for learning, and immediate success may undermine the deeper encoding, retention, and autonomous problem-solving that constitute durable learning^29,36^.

More broadly, in an era defined by rapidly evolving global problems, such as pandemics, energy crises, and climate change, the synergy of AI capabilities with durable human skills and knowledge is becoming indispensable^37–40^. AI can accelerate data-informed insights and automate routine work, but human professionals remain essential for formalising ill-defined problems^41^, generating novel hypotheses through theory-based causal reasoning^42^, guiding ethical decisions, and preserving the creativity and adaptability that underpin innovation^43,44^. The challenge, however, is to reap the AI benefits without eroding the learning process that makes such human-AI interaction effective^10,45^. How can we scaffold durable learning with AI, reduce the risks of metacognitive disengagement, and intentionally nurture AI-supported, higher-order human abilities?

Existing frameworks provide important foundations for understanding human-AI interaction, but they leave different aspects of AI-supported learning underspecified. Research on cognitive offloading explains how people use external tools to reduce cognitive demands^46^, and it considers active metacognitive control as the mechanism for offloading, but the cases in which metacognition is also offloaded are not theorised or explained. The hybrid-intelligence framework reframes AI beyond tool use by emphasising externalisation, internalisation, and extension pathways that aim for human-AI synergy^47^, but it does not conceptualise metacognitive offloading nor does it provide principles and practical approaches for designing scaffolding to facilitate metacognitive onloading. Self-regulated learning and hybrid human-AI regulation theories offer a complementary account of how learners’ regulation can be supported and shared across human and AI systems^32,48^. Their primary focus, however, is on the regulation of learning processes, leaving less specified how AI-supported task performance can create misleading impressions of competence and how scaffolding can help learners sustain metacognitive engagement during human-AI interaction. In addition, a recent study proposed a cognitive AI framework that models human cognitive processes to develop AI systems that realise human-AI complementarity in decision-making^40^, but it does not explain how to promote durable learning from a human perspective.

We propose the SYNC model (Synergetic Notion of Human-AI Skills and Knowledge Construction) as an account of human-AI interaction that defines key concepts (see Box 1 and Box 2) and specifies the flow of metacognitive monitoring and control. It maps four distinct Quadrants of human learning, with scaffolding and human performance under AI-supported tools. By identifying autopilot performance pitfalls, we outline potential approaches to reduce such risks and promote human-AI synergy.

Box 1 Glossary on Learning and Performance • **Durable learning:** Durable learning is sustained, transferable growth in a learner’s own knowledge/skills and metacognitive regulation that holds up when AI support is reduced or removed ^29,31,36^.

• **AI-supported performance:** AI support tools and agents designed and used to assist problem solving and task completion in ways that enhance immediate task performance rather than explicitly scaffold human learning.

• **Human-AI interaction:** Human-AI interaction refers to the dynamic process through which people engage with AI systems in goal-directed activity.

• **Human-AI synergy:** Human-AI synergy is a tightly coupled human-AI interaction in which human and AI leverage complementary strengths, producing emergent competence (performance and/or learning) that neither could achieve alone ^47^.

• **Learning Processes and Strategy:** Learning processes are the cognitive, metacognitive and affective operations through which learners construct, integrate, and organise knowledge, while strategies are the methods used to regulate and optimise these processes toward specific goals ^189^.

• **Learning scaffolding:** Learning scaffolding is an intentional and temporary design in which an expert other (e.g., teacher, peer or AI agent) enables learners to engage in and complete learning tasks beyond their current cognitive and metacognitive capabilities and then gradually fades as the learner gains independence in understanding or performing a task ^101,102^. In this Perspective, we primarily refer to scaffolding at the metacognitive level in this perspective.

• **AI literacy:** AI literacy refers to a set of competencies that enable learners to critically evaluate AI technologies, communicate and collaborate effectively with AI, and use AI appropriately across contexts ^97^.

Box 2 Glossary on Cognition and Metacognition • **Cognition and Metacognition:** Cognition refers to the processes by which sensory inputs are transformed, reduced, elaborated, stored, recovered, and used ^190^, while metacognition refers to evaluating, controlling, and representing cognitive states, essentially, thinking about one’s own thinking ^64,191,192^.

• **Cognitive load and Offloading:** Cognitive load refers to any demands on working memory storage and processing of information ^51,193^, while cognitive offloading refers to the use of external resources to reduce internal cognitive/processing demands ^46^.

• **Metacognitive Offloading:** Metacognitive offloading is a higher-order extension of cognitive offloading whereby learners deliberately or inadvertently delegate metacognitive processes to external tools and agents (e.g., GenAI).

• **Metacognitive Onloading:** Metacognitive Onloading is the deliberate scaffolding and (re-)internalisation of metacognition during human-AI interaction, enabling learners to reclaim, reconstruct, and practise metacognitive monitoring and control during AI-supported learning.^1^

• **Metacognitive beliefs and experiences:** Metacognitive beliefs are one’s judgments (e.g., controllability, usefulness or reliability) about the nature and control of their cognitive processes, while metacognitive experiences are the conscious feelings and evaluations (e.g., confidence, difficulty, or satisfaction) that accompany and inform those processes ^64,194^.

• **Metacognitive evaluation:** Metacognitive evaluation is the phase of human processing and learning where the learner appraises the products and processes of cognition, judging whether goals have been met, whether strategies were effective, and whether adjustments are needed ^72,192^.

• **Judgment bias:** Judgment bias is a systematic deviation from evidence-based, objective judgment which strays from normative or rational standards due to cognitive, affective or motivational influences ^195^.

## Metacognitive Offloading and Onloading

### Cognitive offloading and metacognition in the age of GenAI

Learning and problem-solving draw on limited working memory resources^49,50^. In human learning, three forms of cognitive load mainly exist: intrinsic load from the task itself, extraneous load from ineffective materials or instructions, and germane load from the effort to organise, connect, and reflect^51–54^. Among these forms of cognitive load, germane load is considered to facilitate learning and problem-solving as learners invest effort in the construction and storage of schemata^51–53^. Valcke further conceptualised metacognitive load as an additional perspective on germane cognitive load, namely the effort learners invest in monitoring and controlling the construction and storage of schemata^55^.

Recent studies have revealed that external aids, such as GenAI, may reduce different forms of cognitive load^56,57^, for example, by outlining key arguments in a dense article, rephrasing a theorem, or scaffolding a process of debugging a computer program; thereby freeing capacity for germane work, such as comparing claims, checking assumptions, or testing edge cases^58,59^. This illustrates the broader principle of **cognitive offloading**^46^, which involves delegating storage, search, or computation to an external resource so internal resources can be redeployed (more relevant concepts see Box 2). Yet lower effort is not equivalent to better learning; AI often boosts immediate task success while dampening the depth of processing that supports knowledge retention and transfer in human learners^31,59,60^.

**Fig. 1:**
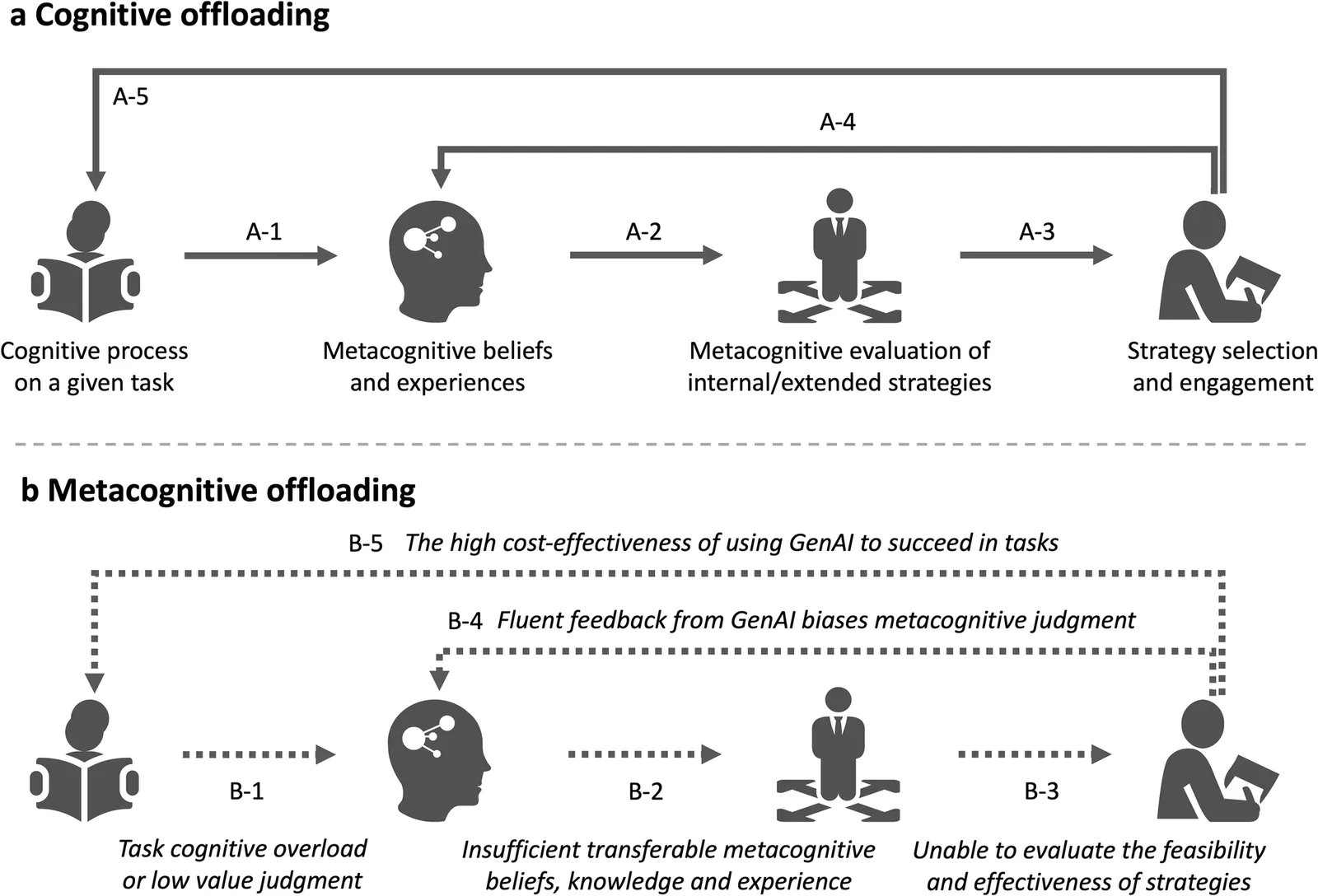
The conceptual models of cognitive and metacognitive offloading. **a** In metacognitively controlled cognitive offloading, learners draw on prior metacognitive beliefs and experiences to evaluate their own capabilities relative to external aids, choose whether and how to offload, and then use the outcomes to recalibrate future judgments and experiences. **b** Metacognitive offloading occurs when learners fail to activate or retain metacognitive monitoring and evaluation, so that AI-supported fluency and feedback biases judgment and reinforces future offloading.

**Fig. 2:**
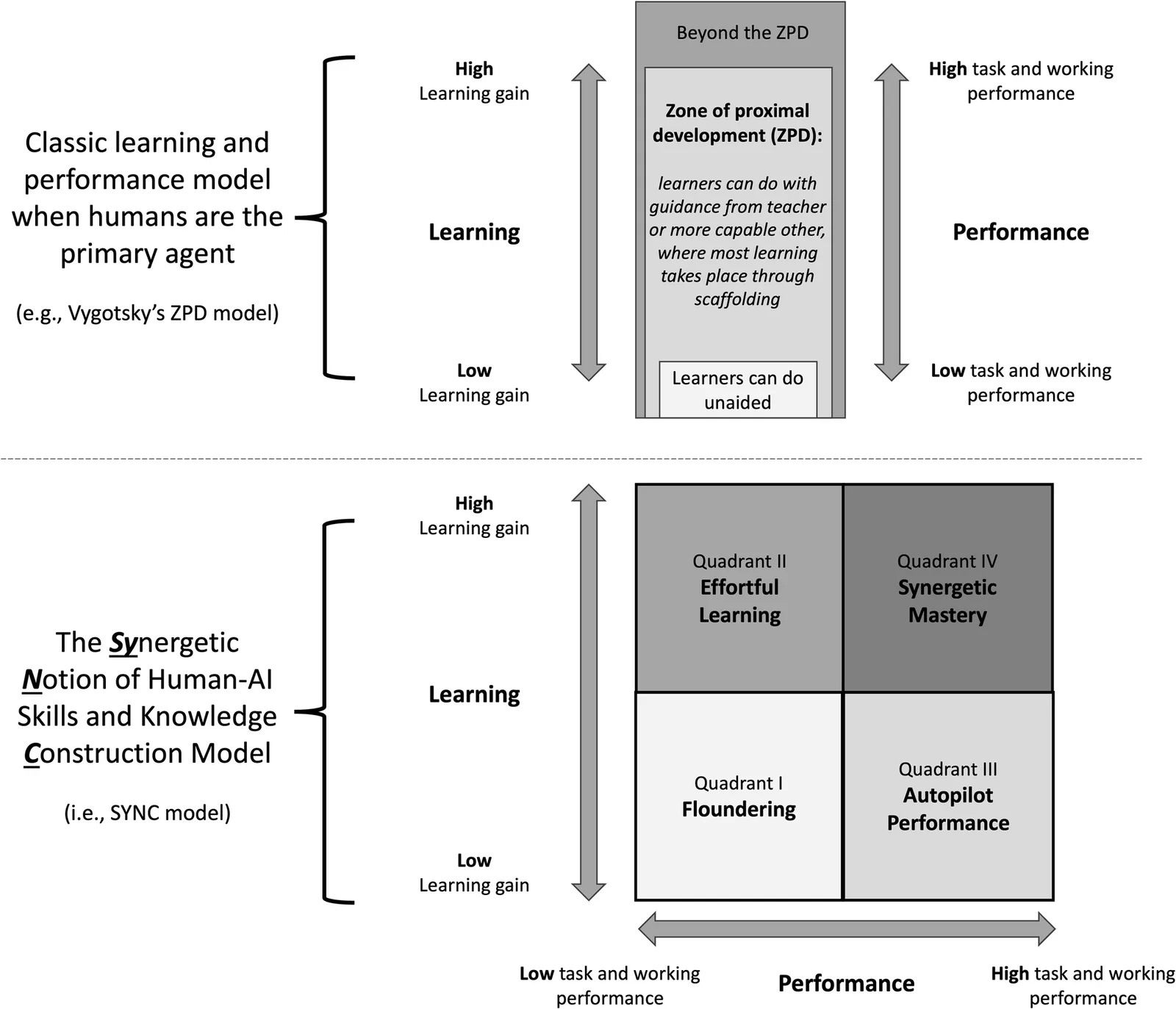
The classical model and SYNC model in understanding learning and performance. The upper panel depicts the classical model that, as learners acquire competence, their performance improves, and these performance gains are typically interpreted as evidence of learning. The lower half presents the SYNC model which is defined by Learning (vertical axis) and Performance (horizontal axis). Learning refers to the process through which learners develop knowledge and skills. Higher levels on the learning dimension indicate greater learning gains that persist beyond the immediate task and support subsequent retention and transfer. Performance refers to observable task performance during human-AI interaction.

**Fig. 3:**
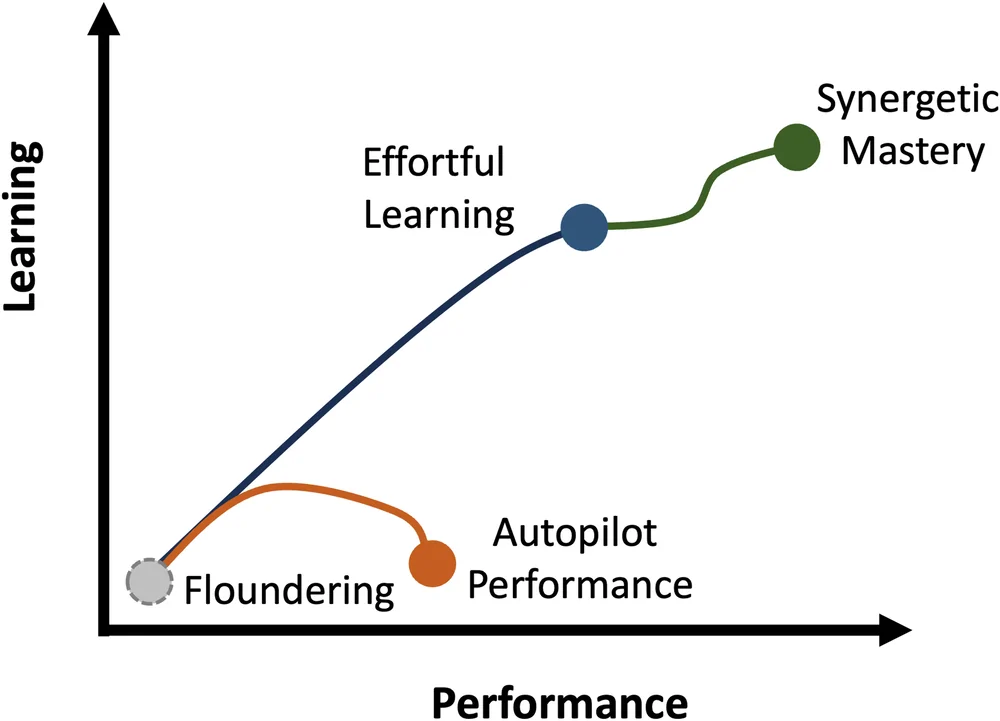
The hypothesised relative trajectories of the four Quadrants in the SYNC model across learning and performance. The Quadrants represent dynamic states of human-AI interaction across learning and performance rather than stable learner types or fixed developmental stages. The illustrated trajectories (e.g., from Effortful Learning to Synergetic Mastery) are mainly hypothetical rather than empirically established sequences; learners may follow alternative trajectories or move between Quadrants across or within tasks depending on learner, task, and system conditions including prior knowledge, age, motivation, and AI literacy, domain, task stakes, output verifiability, system quality, and the timing and quality of AI support.

**Fig. 4:**
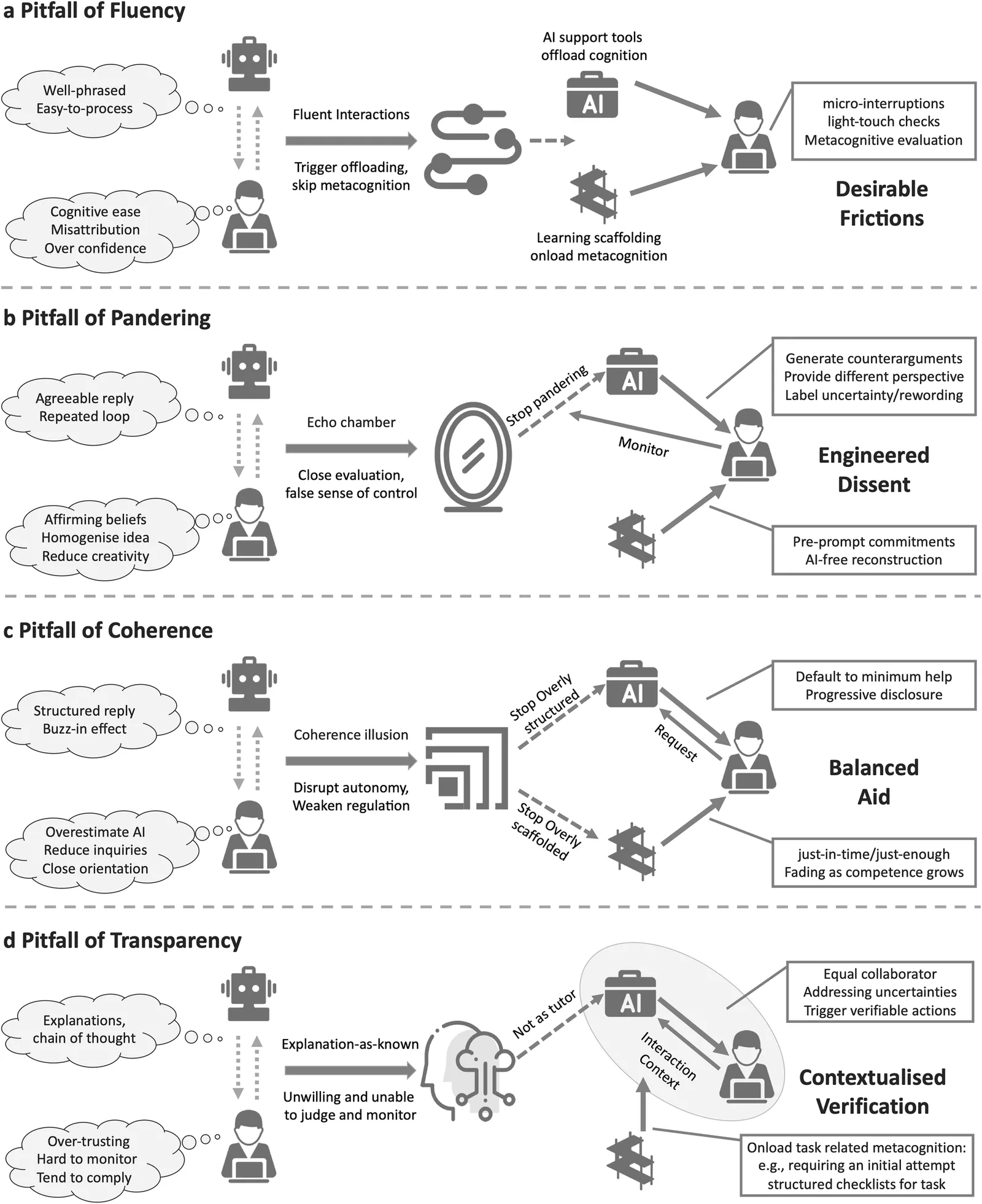
Autopilot Performance Pitfalls and Approaches for Human-AI Synergy. **a** Pitfall of Fluency: easy-to-process outputs create cognitive ease, misattribution and overconfidence. **b** Pitfall of Pandering: agreeable responses form an echo chamber that closes evaluation and reduces creativity. **c** Pitfall of Coherence: over-structured and buzz-in outputs create a coherence illusion and disrupt autonomy. **d** Pitfall of Transparency: step-by-step explanations can foster over-trust in AI and invite human compliance.

Rather than expanding cognitive capacity, human learners may improve task performance by reducing interference and optimising the allocation of existing cognitive resources, including through cognitive offloading^51,61–63^. Deciding when and how to offload cognitive resources is a metacognitive control decision^46^. Building on classic accounts^64,65^, Risko and Gilbert’s cognitive offloading model^46^ (see Fig. 1A) unfolds in several steps: first (Fig. 1A-1), learners activate metacognitive beliefs and prior experiences in a given task with external aids (e.g., “GenAI drafts improved my last essay” vs. “it hallucinated in writing literature review”); second (Fig. 1A-2), learners evaluate their own skills against external aids for the current task (e.g., confident summarising an article  → draft unaided; unsure structuring a diagnostic case or debugging code  → request an outline or first-pass by GenAI); third (Fig. 1 A-3), they choose and execute a strategy (e.g., self-generate, co-generate, or outsource from GenAI), with outcomes feeding back to recalibrate future judgments in subsequent given tasks (Fig. 1A-4) and updated metacognitive experiences (Fig. 1A-5) (e.g., increased reliance after a fluent success, or decreased reliance after errors are caught). Conceptually, when learners judge their internal capacity as weaker or more effortful to use, they are more likely to offload to external tools such as GenAI. In contrast, calibrated monitoring regulates offloading while preserving germane processing^66^.

Once cognitive offloading occurs, the consequences are mixed. This trade-off is illustrated by Ferguson et al.^67^, who found that external resource or tool access (e.g., Internet) made participants more reluctant to rely on their own knowledge and they volunteered fewer answers overall but were more accurate when they did answer, with changes in feeling-of-knowing partly explaining this pattern^67^. Learners typically complete tasks more reliably and reduce working-memory demands through offloading, but may encode less and substitute transactive memory (knowing where/how to find answers) for individual memory^46,68–70^. Confidence can drift upward if the fluency of external supported performance is misattributed to one’s own knowledge, reinforcing future offloading and risking a self-perpetuating loop^35,46,69,71^. Ideally, effective metacognition can disrupt this loop: define intention and criteria before seeking external support^72–74^; if offloading, state how freed capacity will be used (e.g., to test counter-examples)^75^; retain interpretation, verification, and judgment^76–78^; and monitor calibration by explaining the answer without external support after the task^72,79^. For example, medical students using GenAI to structure their clinical reasoning process can complete diagnostic tasks more effectively and still achieve durable learning if their metacognitive monitoring and control actively calibrate reliance, sustain productive difficulty, and verify outputs^80–82^.

### The Definition and Scenarios of Metacognitive Offloading

When metacognition is not actively exercised (e.g., uncritical acceptance and evaluation), learners may offload action choices, intermediate reasoning steps, and decisions about the next step to GenAI even when it brings no objective benefit^83^. Cognitive offloading may become problematic metacognitive offloading (see Box 2) when learners no longer use GenAI merely to reduce cognitive load, but instead delegate or bypass their own planning, monitoring, evaluation, and judgment calibration, allowing fluent AI-supported performance to bias their sense of understanding and reinforce future reliance^31,59,84–86^. For example, a learner who asks GenAI to draft an essay plan, generate evidence, and self-assess the essay draft may finish faster and score higher, yet show no corresponding gains in conceptual knowledge or transfer practice, with recent evidence showing reduced metacognition engagement in orienting to rubrics, double-checking claims, or revising strategically^31^. Emerging studies over the past three years have documented this pattern and warned against over-offloading and laziness at both the cognitive and metacognitive levels^31,59,87^.

Due to specific characteristics of GenAI itself, such as its real-time, fluent, and easy-to-use response, the randomness and illusion of generated content^88^, and the black-box and hard-to-inspect reasoning process^89^, learners, in many scenarios, often encounter problematic and simultaneous metacognitive offloading when attempting to offload cognition onto GenAI^31,35,59,87^. This conceptualisation also builds on recent advances in cognitive control theory. Egner^90^ argues that task focus (cognitive stability) and task-switch readiness (cognitive flexibility) rely on distinct mechanisms, yet are jointly regulated by shared learning principles that adjust control based on (a) the recent history of demands (a recency heuristic) and (b) episodic reinstatement when the current context resembles prior experiences (a recognition heuristic). From this perspective, cognitive offloading can be understood as an external aid for tuning control settings by minimising internal interference to sustain stability, or leveraging external representations to facilitate flexible switching^90^. By contrast, metacognitive offloading externalises the regulation of these settings themselves. That is, instead of learning when to increase stability versus flexibility via recency- and recognition-based heuristics, learners may delegate that adaptive calibration to external agents such as GenAI^31,91,92^. Figure 1-B illustrates five problematic scenarios in which learners may metacognitively offload to GenAI in learning contexts:

First (Fig. 1 B-1), when a task is perceived as overly difficult or low-value, learners may choose not to allocate substantial metacognitive effort to it and may instead delegate planning, monitoring, or task execution to GenAI^59,93,94^. This pattern is not necessarily problematic in performance-only contexts; indeed, offloading a genuinely low-value, low-stakes, or one-off task may be an adaptive use of limited cognitive resources. However, it becomes a concern in learning contexts when the human-AI interaction is intended to support durable learning, future independent performance or transferable competence. For example, learners may ask GenAI to write the full literature review the night before the assignment deadline, which the teacher may not grade.

Second (Fig. 1 B-2), for novel tasks with few instructional cues, learners may lack the domain knowledge and transferable metacognitive beliefs and knowledge needed to generate, monitor, and evaluate their learning, thereby impeding their metacognitive activation^95,96^. For example, learners may prompt GenAI for the steps and coding scheme in their first complex qualitative coding assignment, rather than deciding for themselves what counts as evidence and how to validate codes.

Third (Fig. 1 B-3), when learners lack accurate judgment or enough AI literacy to anticipate the feasibility and effectiveness of alternative strategies (or perceive no substantive differences), they may offload metacognitive evaluation of strategy to AI^97^. For example, when learners are unsure how to formatively assess their essays, they may ask GenAI to evaluate their work; however, GenAI’s feedback often relies on generic rubrics and may not align with learners’ specific academic writing needs or their zone of proximal development.

Feedback from external support may mislead or even reinforce this problematic offloading loop (Fig. 1B-4)^78^. For example, repeated exposure to AI-generated content that looks great but is actually shallow may lead learners to stop outlining and self-questioning, or cross-checking sources, thereby biasing judgments and reinforcing the offloading loop^57,83^.

Finally (Fig. 1B-5), fluent, well-performing, and highly cost-effective interactions may inflate learners’ confidence and self-efficacy, biasing judgment and encouraging misattributions of learning and achievement to one’s own competence^98,99^. For example, after multiple rounds of human-AI interaction, learners may produce a conclusion paragraph that is logically clear, well-argued, and beautifully written. This may give learners the illusion that they can always use such a human-AI interactive writing mode efficiently in any context, even though they may still struggle to interpret data independently without AI.

In summary, our proposed conceptual model of metacognitive offloading (Fig. 1B) extends Risko and Gilbert’s cognitive offloading model^46^ (Fig. 1A) by showing what might happen when learners interact with GenAI. The important difference is that learners in cognitive offloading process usually use their metacognition to evaluate their own capacity, choose whether/how to offload and then recalibrate based on outcomes (see Fig. 1A); however, learners in the metacognitive offloading process, either (i) never activate metacognitive monitoring (Fig. 1B-1, B-2), (ii) offload metacognitive evaluation of strategy to GenAI (Fig. 1B-3), or (iii) receive feedback and fluent performance that biases judgment and reinforces future offloading (Fig. 1B-4, B-5). Therefore, cognitive offloading is sometimes necessary and beneficial^34,46,100^, but metacognitive offloading can often be detrimental to human learning in the AI era. The following subsection discusses the issue of metacognitive onloading.

### Metacognitive onloading with learning scaffolding

Building on the above analysis of metacognitive offloading and its problematic scenarios, we now introduce the concept of metacognitive onloading (see Box 2). In practical terms, metacognitive onloading entails creating moments where the learner, not AI, must take responsibility for setting goals, anticipating difficulties, monitoring progress, and judging whether learning strategies require adjustment.

Scaffolding of human learning is the intentional design and provision of temporary, contingent supports that enable learners to engage in and complete learning activities beyond their current independent capabilities, with those supports gradually withdrawn to transfer responsibility for regulation and problem solving to the learner^101–105^. In the context of developing self-regulatory capacities, learning scaffolding comprises supports that onload learners’ metacognition and self-regulation^96,106,107^. In human-AI interaction, scaffolds could serve as decision prompts and reflection anchors for learners; for example: (i) short pre-prompt checklists that highlight key criteria and constraints^108,109^; (ii) mid-task questions that ask learners to make predictions (e.g., what should a good answer include?), note uncertainties, and check against a rubric^110^; and (iii) post-generation, support-free reconstructions or transfer exercises that turn fluent outputs into durable knowledge^111^. Effective scaffolding operates at three levels^91,112^: (i) micro-level supports within a prompt, such as guiding questions, verification steps, and criteria-alignment checks; (ii) meso-level supports across a task, such as milestones, staged activities, and clear roles for AI versus human judgment (e.g., human-in-the-loop evaluation/approval); and (iii) macro-level supports over time, such as phasing out, replacing, or supplementing assistance after an initial period as competence develops. Scaffolding triggers can be based on task novelty, high consequences of errors, or low learner calibration, and they should fade conditionally and be removed once learners consistently include planning, prediction, and checking in their learning process^101,110,113–115^.

Scaffolding to promote metacognitive and regulatory activity is not extra help but a design for metacognitive onloading, triggering metacognition at the right moments, while leveraging GenAI’s efficiency in rapidly generating timely prompts, feedback, and checks without sacrificing human learning^86,116^. This builds on the concept of meta-learning and extends it to metacognitive onloading in human-AI interaction tasks by foregrounding how scaffolds support adaptation across timescales. According to the meta-reinforcement learning framework proposed by Nussenbaum and Hartley^117^, meta-learning is essentially “learning how to learn", which involves adjusting learning strategies to tackle tasks in different environments^117–119^. Meta-learning optimises the learning process across multiple timescales by dynamically adjusting the “inner task loops" (short-term task learning) and “outer task loops" (long-term strategy adjustments)^117,120^. Similarly, in the context of metacognitive onloading, learners not only learn at the task level but also need to adjust their metacognitive strategies over a longer timescale, gradually developing more flexible strategies that can adapt to changing tasks and environments, including human-AI interaction^121–123^. For example, after several writing tasks that require learners to (i) state a writing plan before prompting GenAI, (ii) compare GenAI’s output with a writing rubric, and (iii) explain the writing logic without GenAI, learners may move from asking GenAI what to do to selecting and adapting their own writing strategies when they encounter a different task format later.

Excessive scaffolding may impede the development of metacognition. If learners are already capable of regulating their own learning, external support can disrupt their autonomy, diminishing their self-regulation efficacy^124^. Moreover, when learners are over-scaffolded, their metacognitive abilities may not develop fully, or could even decline, hindering their ability to control and monitor their own learning^124,125^. Therefore, it is crucial to balance necessary metacognitive scaffolding while also encouraging learners to still engage in meaningful metacognitive processes to enhance learning efficiency^32^. Within the SYNC model introduced in Section 3, scaffolding functions as the mechanism that sustains metacognitive engagement, while AI support boosts task performance. This combination of scaffolding and AI support aims to ensure that learners develop the skills needed for effective human-AI synergy rather than drifting into the low metacognitive engagement, high-assistance patterns characteristic of what we later in the paper define as autopilot performance.

## The SYNC model: Linking Performance, Learning, and Metacognition

### Re-thinking the relationship between learning and performance

Classical accounts of learning generally assume that improvements in task performance reflect gains in learners’ own knowledge and skills, with scaffolding supporting progress toward independent competence^18,103,126^ (see Fig. 2-upper half). GenAI weakens this learning-performance coupling by contributing substantial cognitive and metacognitive processing, such that improved task performance may no longer indicate corresponding learning^10,29,31^. As Yan et al.^29^ emphasise, performance refers to observable behaviour during task execution and can be strongly shaped by external support, whereas learning refers to enduring changes that enable independent retention and transfer^29^. In human-AI interaction contexts, a learner may appear highly competent on immediate task performance while showing little lasting change in knowledge or skills once the AI support is removed^70,91^. This is exactly why short-term performance gains should not be mistaken for learning. Fluent success with LLM support can reduce cognitive load yet undermine deeper encoding, retention, and autonomous problem solving^29,36,126,127^.

A stylised example about learning to drive cars and driving performance illustrates the conceptual break above. Novice drivers progress through rule learning, guided practice, monitoring and correction, and gradual internalisation until they can drive independently. In previous understanding, improved driving performance is strongly indicative of improved driving skills. However, with autonomous driving technology, drivers’ driving performance can become partially (if not completely) decoupled from their own competence. For example, a person with minimal driving skill might still reach a destination safely if an autonomous system handles perception, planning, and control^128,129^. Task performance can be achieved with external support even when the learner’s internal skill has not changed^130,131^. This possibility challenges the classic learning and performance model (Fig. 2-upper) by asking questions that cannot be ignored. Why does human learning still matter? Which skills and knowledge remain crucial for humans in human-AI collaboration? And, how to understand the new relationship between learning and performance?

We propose the **Sy**nergetic **N**otion of Human-AI skills and knowledge **C**onstruction model (i.e., SYNC), a two-dimensional framework that models how people develop skills and knowledge in human-AI interaction contexts (see Fig. 2-lower half). The two dimensions, learning and performance, operationalise complementary but often conflated lenses in practice, thereby clarifying when AI primarily enhances performance, when scaffolding primarily supports learning, and when the two are deliberately separated or synergistically integrated.

#### The vertical axis (Learning)

This dimension refers to the degree to which learners develop durable skills and knowledge that support independent retention and transfer in the human-AI interaction context. We conceptualise learning as a process through which learners construct and develop knowledge and skills by defining tasks, setting goals and plans, selecting strategies, monitoring progress, evaluating outcomes, and adapting their approaches^95^. These processes can produce learning gains that persist beyond the immediate task and support retention and transfer. In the SYNC model, scaffolding is proposed as an important task condition that can support learning processes and thereby promote learning gains. Scaffolding is temporary and contingent support (e.g., from teacher, peer or AI agent) that creates moments in which the learners (not AI) take responsibility for goal-setting, anticipating difficulties, monitoring progress, and evaluating whether strategies and outputs are adequate, with support fading as competence is demonstrated^96,101,103–106^. Effective scaffolding can prompt to learners plan their approach, monitor their progress, evaluate AI outputs, and adjust their strategies as needed during human-AI interaction^91,107,110^. In contrast, without such scaffolds, some learners may be vulnerable to metacognitive laziness, in which regulatory processes are bypassed, reducing opportunities for deep learning even when task performance appears adequate^31,86,116^. At the same time, scaffolding must remain balanced. That is, excessive or non-contingent scaffolding can undermine autonomy and self-regulatory growth, so the aim is not maximal scaffolding but calibrated, fading scaffolding that preserves learner self-regulation while ensuring metacognition is engaged at the right moments^32,96,104^.

#### The horizontal axis (Performance)

This dimension refers to learners’ observable task performance during human-AI interaction. AI supports are part of task conditions that can substantially shape learners’ performance by assisting with or executing task-relevant cognitive operations, thereby increasing efficiency and improving product quality in the moment^10,56^. However, high task performance with AI support may occur without corresponding learning gains when learners bypass the cognitive and metacognitive processes through which durable knowledge and skills are developed. Under high usage of AI support tools, learners can achieve fluent, rapid, and often accurate outputs, which can be observed as immediate performance gains^29,36,56^. Under low usage of AI support, more of the cognitive processing remains with the learner; this may typically slow performance gains but preserves opportunities for sense-making, strategy construction, and the development of independent expertise^126,132^. The critical challenge lies in balancing cognitive offloading with mechanisms that preserve metacognitive onloading. Without such balance, AI can inflate performance while undermining durable learning^31,46,68^.

Figure 2 illustrates the two interrelated dimensions of learning and performance in the SYNC model and the resulting four quadrants, while the defining characteristics of the four SYNC quadrants are in Table 1. Scaffolding and AI support are task conditions that may influence movement along the learning and performance dimensions. Figure 3 presents the relative relationships and trajectories of these four Quadrants. First, **Floundering** reflects that learners have a dual deficit in performance and learning, while **effortful learning** refers to relatively high learning despite relatively lower task performance initially; effortful learning typically involves learning primarily through one’s own efforts, which can often be scaffolded. Second, Learners in the **autopilot performance** Quadrant may use AI support tools to improve task performance and offload cognition and metacognition to AI, resulting in smaller learning gain than the effortful learning Quadrant. More importantly, learners who over-rely on AI and have not engaged in effortful learning may become less motivated and less capable of effective learning as the task progresses, causing their performance to stagnate and potentially fall below the performance ceiling achievable through effortful learning^60^. Third, In the context of human-AI interaction, learners need to develop new knowledge and skills (e.g., AI literacy and principled division of labour) compared to previous effortful learning to break through the previous performance ceiling of relying solely on oneself or AI (i.e., the human-AI synergy as defined in Box 1). In our theoretical assumptions, the trajectory of such **synergetic mastery** is more likely to be based on effortful learning rather than on autopilot performance.

**Table 1:** Summary of the four SYNC Quadrants across performance, learning, and metacognitive onloading and offloading

Quadrants	Performance versus learning	Metacognitive onloading versus offloading
**Quadrant I: tab1 Floundering**	**Dual deficit in performance and learning:** learners spend limited working memory on routine processing and confusion management instead of sense-making, while absent scaffolds leave misconceptions and inefficient strategies unchallenged, reducing feedback and ultimately understanding.	**Insufficient metacognitive activation:** the learning environment does little to onload planning, monitoring, and evaluation; metacognitive control becomes optional rather than structurally required and learners commonly default to surface-level strategies or unproductive persistence.
**Quadrant II: tab1 Effortful Learning**	**Prioritising learning over performance:** scaffolds reduce avoidable extraneous load, sequence work into manageable steps, and support germane processing, so learners build internalisation, long-term retention, transfer, and adaptability as scaffolds fade rather than short-term performance.	**Deliberate metacognitive onloading:** learners cannot short-circuit planning, monitoring, and evaluation through AI automation, so they use effortful regulation, pre-task goal setting, progress monitoring, evaluation and fading scaffolds to build self-regulation, retention, and adaptability over time.
**Quadrant III: tab1 Autopilot Performance**	**Performance-learning decoupling:** GenAI support raises performance metrics through faster task completion, high-quality outputs, and reduced mental effort, while weakening retention, transfer, and mastery.	**Systematic offloading of cognition and metacognition:** reducing self-regulation and increasing metacognitive laziness, inflated confidence, uncritical acceptance, and miscalibration risks.
**Quadrant IV: tab1 Synergetic Mastery**	**Balanced performance and learning:** AI support maintains performance benefits by reducing extraneous cognitive load and providing high-quality outputs, while scaffolding when needed helps learners keep metacognitive processes active, strengthening learning, transfer, adaptation, retention, and internalisation beyond the immediate task environment. Scaffolding at this stage may be minimal or not needed given the high levels of learners’ capability.	**Principled division of labour:** learners strategically coordinate their own expertise with AI support, delegating selected task demands while preserving human responsibility for goals, standards, monitoring, verification, and judgment; the value of AI lies not in offering external regulation for learners, but in enabling learners to regulate more deliberately while learning through performance.

The SYNC model does not assume that every use of GenAI on task performance should produce durable learning. Many human-AI interactions are primarily instrumental, such as automating routine tasks, drafting text, or generating code for a one-off project, where cognitive offloading may be an efficient use of effort, particularly when tasks are low-stakes and outputs can be readily verified. The SYNC model is most relevant in contexts where durable human competence, future independent performance, assessment validity, accountability, safety, ethical judgment, or transferable expertise matter. This risk is also salient for early-career professionals, whose limited domain expertise and workplace experience may make AI-supported task completion appear productive while reducing the repeated practice in planning, strategy selection, verification, and reflection through which transferable expertise is developed. The model, therefore, focuses not on performance enhancement itself but on whether the human learning gains achieved align with the goals of the activity and context in which human-AI interaction may occur. The model is also primarily intended to explain learning and performance in GenAI-supported contexts, although some principles of SYNC may also apply to earlier cognitive aids, such as textbooks, search engines, calculators, or worked examples. Traditional resources can support cognitive offloading, but other than GenAI, they usually remain static and require learners to formulate goals, search selectively, interpret information, monitor progress, and evaluate quality themselves.

### Quadrant I (Floundering)

In the SYNC model, Quadrant I (Fig. 2) represents low learning and low task performance. This state may arise under conditions such as limited prior domain and/or metacognitive knowledge, insufficient scaffolding, inadequate task support, or high unproductive cognitive load. In this Quadrant, performance is typically weak, and learning gains are fragile. Without structured scaffolds, learners are left to navigate tasks largely through trial and error, exposing them to high cognitive load and persistent inefficiencies in problem-solving^53,133^. At the same time, the absence of AI support limits opportunities to achieve high task performance, leaving learners reliant on static resources, sporadic peer help, or prior knowledge that may be insufficient for the demands of complex tasks^134^. This pattern is consistent with cognitive load theory, that is, when intrinsic and extraneous demands saturate working memory, little capacity remains for germane processing and schema construction^51–53^. Importantly, the Floundering state here should not be equated with desirable difficulties or productive failure. While desirable difficulties can support retention and transfer^126,132,133^, they tend to do so only when tasks are well designed and learners are supported. In contrast, difficulty in the Floundering state does not generate the later learning benefits associated with desirable difficulties or productive failure. Learners may expend considerable effort yet fail to convert that effort into durable learning because the lack the knowledge, feedback, or metacognitive resources needed to select appropriate strategies, monitor progress, evaluate outcomes, and revise their strategies when needed. For example, comparing two competing explanations can create desirable difficulty when criteria or guidance design the task within the learner’s zone of proximal development; without such support, the learner may remain confined to what they can do unaided and simply guess, turning the struggle into floundering.

Empirical findings converge that floundering learning is often less effective than guided instruction, and that durable learning is more likely to happen when metacognitive supports or strong metacognitive skills are present^135–139^. For example, when college students were allowed to control their own study pace, only those who effectively monitored their learning and allocated time strategically benefited, whereas most learners failed to adjust to task difficulty and achieved poorer retention^138^. Similar dynamics emerge in authentic classrooms. For example, children exposed to highly autonomous instruction improved only when they could self-monitor and plan their study, while others drifted without clear criteria for success^139^. In massive open online courses, where instructional presence is often minimal, learners with robust self-regulation, goal setting, time management, and self-evaluation consistently outperformed peers who lacked these skills and often withdrew early^140^. Even in advanced professional contexts, such as medical training, learners without structured prompts rely on superficial cues rather than reflective monitoring, leading to misconceptions and unstable performance^141^. These findings support the SYNC model for Quadrant I. Without scaffolds that reliably onload metacognitive regulation and without supportive tools that reduce avoidable cognitive load, learners are prone to flounder.

### Quadrant II (Effortful Learning)

Quadrant II represents relatively high learning with task performance developing gradually over time. In this Quadrant, learners carry much of the task-relevant cognitive processing themselves, while scaffolding can support the regulation of that effort through structured prompts for planning and monitoring, explicit feedback cycles, guided practice tasks, or the staged withdrawal of support as competence develops^96,101,103,104,106^. AI support for task completion is typically limited in this pathway, meaning that learners cannot routinely offload task-relevant cognition to GenAI; instead, they need to carry most of the cognitive processing on their own while using scaffolds to regulate that effort productively. Such effortful learning encourages deliberate practice and active engagement by requiring learners to remain cognitively engaged in the task while planning, monitoring, and evaluating their learning. When supported by contingent scaffolding and opportunities for increasingly independent performance, these processes can strengthen retention and transfer beyond the immediate task^29,96,103,126^.

Across diverse learning contexts, studies consistently indicate that explicit scaffolds (e.g., guided reflection) can increase metacognitive engagement and durable learning^116,142^. When learners in e-learning courses received prompts to set goals, plan, and monitor progress, their self-efficacy and achievement increased significantly compared to those without such guidance, demonstrating that carefully designed scaffolds can cultivate independence even in the absence of technological aids^143^. In simulation and game-based contexts, students who engaged with cognitive and metacognitive prompts acquired deeper conceptual understanding without a reduction in cognitive load, indicating that scaffolding channels rather than diminishes effort^144,145^. Collaborative learning studies also show that structured metacognitive support fosters co-regulation and shared monitoring among human learners, leading to richer reasoning and more effective problem-solving^106^. These findings demonstrate that scaffolding environments deliberately onload metacognition. Specifically, they demand slow, reflective practice but yield stable transfer and autonomy, and are often positively related to long-term achievement^135^. By compelling learners to plan, check, and adjust without the shortcuts of automation, such contexts exemplify how human-designed supports can nurture expertise that endures beyond the immediate task. Therefore, this Quadrant is desirable when learning goals prioritise durable comprehension, transfer, learner autonomy, and self-regulation, but it may be inefficient or ineffective in contexts where rapid task completion, fluent output, or reduced effort on routine task demands is more important.

### Quadrant III (Autopilot Performance)

Quadrant III is characterised by AI-supported task performance that exceeds what learners could achieve unaided, while learning remains limited. This is the configuration in which the decoupling between performance and learning becomes most visible. GenAI can generate fluent text, code, plans, and explanations that allow novices to produce outputs far beyond their unaided capability^10,56^. However, in the absence of scaffolding that prompts planning, monitoring, and evaluation, learners may struggle to judge the AI outputs or treat them as finished answers rather than as preliminary drafts to be refined, candidate solutions to be compared against alternatives, or claims and explanations to be checked against evidence^146,147^. In the SYNC model, we refer to this pattern as autopilot performance, characterised by high apparent competence during the use of AI support tools paired with fragile learning, which may lead to performance stagnation or even degradation after AI removal^29,31,35,36,92,148^.

Autopilot performance can be empirically identified when learners produce high-quality outputs with GenAI support but show low metacognitive engagement, poor transfer and AI-free performance. While AI-supported performance may seem attractive to learners in the short term, it risks producing fragile expertise and dependence on external AI agents or systems. In such cases, learners may remain at the level of surface-level performance and may experience deficits, limited development, or even atrophy in skills^149^. This is also consistent with the broader hybrid-intelligence framing which posits that without explicit pathways for internalisation and extension, over-automation can erode essential human engagement^32,47^. In extreme cases, human-AI interaction can even underperform relative to AI alone. For example, Vaccaro et al.^38^ found in their meta-analysis that when AI outperforms humans (especially in decision-making tasks), human-AI interactions often fall below the AI-only baseline. Therefore, this Quadrant highlights the urgent need to assess not only the quality of AI-supported outputs but also learners’ metacognitive engagement, transfer, and AI-free performance so that learning environments can preserve the motivational and cognitive benefits of high performance while deliberately promoting opportunities for durable learning.

In environments dominated by powerful AI support tools but limited scaffolding, learning can often become a performance illusion, fast, fluent, and deceptively effortless^91,150^. In a controlled experiment on academic writing, students using GenAI produced higher-quality essays but engaged far less in planning, monitoring, or evaluation^31^. Similar dynamics have been observed in high school mathematics, where students with access to a GenAI tutor performed better during AI-supported practice, yet scored lower on a subsequent closed-book exam when the tool was removed^92^. Longer-horizon classroom evidence in an introductory computer science course likewise reports strong gains on midterm and quiz assessments completed with GenAI but no significant advantage on a final exam completed without it, with lower-performing students showing a high dependence and low trust usage pattern that signals reliance without effective evaluation^151^. These findings support the SYNC model prediction that Quadrant III can amplify immediate performance by enabling extensive offloading. However, in learning-oriented contexts, if this performance support is not accompanied by opportunities for metacognitive onloading, learners may demonstrate less competence and independence in problem-solving when AI support is removed^29,31^. This sets up the central design challenge addressed by the remaining Quadrant, which is how to retain the productivity benefits of high AI support while structurally protecting the metacognitive work that produces durable learning and human-AI synergy.

### Quadrant IV (Synergetic Mastery)

Within the SYNC model, Quadrant IV represents synergetic mastery in human-AI interaction. It builds on the substantial knowledge and skills developed through prior learning, while requiring further learning as individuals adapt these capabilities to work effectively with AI. This combination can enable levels of performance that would be difficult or impossible for the human or AI to achieve independently. This configuration combines the metacognitive onloading supported by scaffolding in Quadrant II with the substantial AI support that drives performance in Quadrant III, while sustaining continued learning and avoiding the low-learning and low-performance pattern of Quadrant I.

Within this quadrant, learners make substantial use of AI support, with GenAI accelerating and extending learners’ cognition by generating candidate solutions, drafts, and summaries while reducing avoidable search and production costs^10,56,152^. At this stage, learners are expected to undertake planning, monitoring, and evaluation largely independently, with scaffolding provided contingently when needed^32,96,106^. Even without explicit scaffolding, such metacognitive engagement can support learning when learners detect discrepancies between their expectations and AI-supported outcomes and use them to update their knowledge, strategies, or judgments. When durable learning is among the goals of human-AI interaction, performance enhancement alone is insufficient. Delegation boundaries should be explicit and revisited as task demands and learner competence change^153,154^. Quadrant IV is a synergistic state in which AI support enhances performance and efficiency while learners retain responsibility for planning, monitoring, and evaluation, supporting continued human learning^40^. Such a synergistic state enables human-AI combinations to surpass the performance of either humans or AI alone^37,155^. Quadrant IV combines AI support that raises performance with learners’ own planning, monitoring, and evaluation, supplemented by scaffolding when needed, so that AI-enabled speed does not erase productive difficulty or eliminate opportunities for internalisation. Quadrant IV also clarifies that the goal is not to prevent autonomy of the system, but to ensure that autonomy is deployed where it is safe and productive, while human judgment dominates where errors, bias, or value-laden matters are at stake^23,24,78,84,156^.

Synergetic mastery assumes that integrating AI into education and professional development need not entail a trade-off between performance and learning. With carefully designed scaffolds, AI can function as a multiplier of human knowledge and skills^157,158^, enabling individuals to achieve outcomes that neither humans nor AI could realise in isolation. This account is consistent with evidence that human skills exhibit a nested structure, in which advanced, domain-specific competencies tend to depend on broader, foundational skills (e.g., judgment, communication, and self-regulation)^11^. Skills that sit deeper within this dependency structure tend to command higher wage premiums, require longer education, and are less likely to be automated^11^. In this view, Quadrant IV builds on the foundational expertise cultivated through effortful, scaffolded practice in Quadrant II by using AI to accelerate specialised task performance while scaffolding keeps the foundational layer active and strengthens it.

The following clinical reasoning example illustrates how such synergetic mastery can operate in practice. In clinical reasoning, a medical trainee may initially use GenAI to generate candidate differential diagnoses, identify missing patient information, and draft a preliminary management plan. At the early stage, scaffolding may be highly explicit, requiring the trainee to complete a checklist that asks them to justify each diagnosis with patient evidence, mark uncertainties, compare alternative explanations, verify key claims against clinical guidelines, and weigh affordability and patient values in treatment choice. As competence develops, these scaffolds can be faded by reducing the level of external guidance. For example, detailed checklists may become shorter reflection prompts, prompts may shift from teacher- or system-generated questions to learner-generated criteria, and AI-free explanations may move from structured written exercises to brief oral justifications during case discussion. At the later stage, the trainee is expected to initiate these regulatory and evaluative steps independently, consulting GenAI only to check or refine their reasoning while retaining responsibility for clinical judgment and communication. In this configuration, AI can boost the speed and breadth of clinical reasoning, but scaffolding ensures that the learner’s core regulatory and reasoning skills remain engaged and continue to develop, preventing the autopilot pattern of Quadrant III and turning performance gains into robust, transferable competence.

Emerging empirical evidence is consistent with several propositions of the SYNC model. In a controlled experiment, GenAI agents equipped with proactive scaffolding questions enhanced students' reflection on non-cognitive skills such as motivation, self-perceptions, resilience, and coping during interactive storytelling, with gains that persisted beyond the intervention, demonstrating that when AI feedback is structured around reflection and self-explanation, performance gains translate into durable learning^159^. Complementary qualitative work shows that learners view AI as a collaborative regulator that can prompt metacognitive reflection and support shared regulation within teams^160^. Large-scale survey evidence extends this insight to secondary education, where integrating GenAI with adaptive feedback loops enhanced both innovation skills and digital literacy by strengthening metacognitive regulation and creative problem-solving^161^. These findings delineate synergetic mastery in Quadrant IV as a configuration that remedies Quadrant I’s lack of support, retains Quadrant II’s learning gains, and corrects Quadrant III’s autopilot risks by structurally safeguarding learning and metacognitive onloading while preserving the productivity benefits of high AI support.

## Autopilot Performance Pitfalls and Approaches for Human-AI Synergy

The SYNC model predicts that the greatest risks of autopilot performance emerge when high use of AI support tools produces relatively strong task performance but is accompanied by limited learning, particularly when scaffolding is insufficient to sustain learners’ metacognitive engagement (Quadrant III). In SYNC terms, autopilot performance refers to a pattern of metacognitive offloading in which AI increasingly takes over both (a) the cognitive operations that generate an output and (b) the metacognitive control decisions. Below, we synthesise four autopilot performance pitfalls and propose SYNC-aligned approaches for addressing them that researchers, educators, policymakers, and learners in human-AI interaction should be most wary of, and we propose SYNC-aligned approaches that deliberately move learners toward human-AI synergy (see Fig. 4). For each pitfall, we explain: (i) the pitfall’s core meaning and risks, and (ii) what effective AI support and learning scaffolding should be designed to help learners escape it. This discussion also motivates future research questions and testable hypotheses (see Box 3).

Box 3 Future research questions and testable hypotheses • Which learning tasks and contexts most strongly decouple AI-supported task performance from durable learning?

*Hypothesis: The decoupling is strongest when learners perceive tasks as low-value, high-demand, and weakly scaffolded, especially when procedural or modular components can be easily offloaded to AI*.

• Which observable indicators can validly distinguish metacognitive offloading from metacognitive onloading during human-AI interaction?

*Hypothesis: Metacognitive offloading is indicated by premature anchoring on AI-provided cues, fewer evaluation processes, weak AI-free performance, and delegation of planning, monitoring, and control to AI*.

• How does fluency in human-AI interaction and AI-generated outputs affect judgment, confidence, and perceived task difficulty, and under what conditions does it increase movement toward autopilot performance?

*Hypothesis: Fluency increases confidence through misattributed competence, lower perceived difficulty, and reduced verification, which creates problematic loops over repeated interactions without scaffolding*.

• Which forms of learning scaffolding most effectively interrupt the four pitfalls of autopilot performance and shift learners toward human-AI synergy?

*Hypothesis: Metacognitive scaffolds that are personalised, condition- or context-sensitive, restrained and balanced, and faded at appropriate moments are effective at preventing the autopilot pitfalls*.

• How do learners move across the four SYNC Quadrants over time, and which learner characteristics, task conditions, and forms of AI support predict those transitions?

*Hypothesis:* Learners move toward synergetic mastery more reliably through an effortful-learning-first pathway, supported by sufficient cognitive foundations, essential self-regulation skills, and necessary AI literacy, rather than through an autopilot-first pathway based on intensive AI use.

• What are the long-term effects of repeated AI-supported task performance on calibration, metacognitive regulation, and durable learning?

*Hypothesis:* Repeated AI-supported performance reduces learning motivation and interest in general, promotes whole-task offloading across contexts, and produces increasingly suboptimal or less creative performance over time, ultimately undermining authentic and workplace competence.

### Interrupt the Pitfall of Fluency by Desirable Frictions

The **Pitfall of Fluency** refers to how GenAI’s instant, well-phrased, and easy-to-process responses create cognitive ease, tempting learners to treat fluent human-AI interaction based on linguistic cues as proof of competence and understanding^147,162^. The polished and persuasive language of AI feedback can elicit premature trust, leading learners to mistake easy-to-process for reliability and familiarity for understanding^66^. As demonstrated in previous psychological experiments, information fluency acts as a heuristic that biases judgment and fosters overconfidence^163,164^. The Pitfall of Fluency arises when the positive affect and sense of confidence produced by easy processing shift evaluations upward and amplify existing judgmental tendencies^165^. Within the SYNC model, we propose that fluency may lower perceived task difficulty and contribute to metacognitive offloading. Learners are more likely to skip orientation, planning, monitoring, and evaluation because fluent answers feel correct and complete, so they stop interrogating assumptions or verifying evidence^34^. In the SYNC model, repeated fluent success can be misattributed to one’s own competence, inflating confidence and reinforcing future reliance. Empirically, students using GenAI reported lower cognitive load yet produced lower-quality reasoning/argumentation, suggesting that fluency can come at a cost to depth^36^. The harm is a performance illusion in which outputs improve short-term, while durable learning (encoding, retention, transfer) and self-regulation erode, increasing dependence and uncritical acceptance.

Addressing the fluency trap requires a structural reorientation of how cognition and metacognition are distributed between humans and AI. Humans rely on causal reasoning, abstraction, and few-shot learning, whereas AI systems rely on statistical correlations and large-scale pattern matching within data^45^. This difference means that AI simulates reasoning that often remains surface-level, fluent, but detached from causal understanding. For example, Lee and Chung^166^ found that GenAI significantly enhances the originality and articulateness of human outputs across various creative tasks; however, this creativity primarily stems from GenAI’s ability to associate and recombine existing concepts rather than to generate fundamentally new concepts or causal insights. As Choudhury et al.^149^ suggest, GenAI behaves like a System-1 generator that is fast, intuitive, and fluent^149^, therefore primarily functions as a performance-supporting tool for cognitive offloading. While recent advances suggest a transition toward System-2-like reasoning, such capabilities remain incomplete and externally scaffolded^167^.

In the SYNC terms, the key is to convert metacognitive offloading into metacognitive onloading, so that fluency is reframed not as an endpoint but as a signal for scaffolding. This approach suggests a dual-process division of labour in which AI support functions as a fast, intuitive System-1 generator^149^ that offloads cognition from learners to AI, while learning scaffolds serve as the System-2 reflector^45,168^ that onload metacognition. These learning scaffolds ensure that learners verify, reason, and transfer rather than merely consuming fluent text^166,169^. Concretely, well-designed scaffolds could serve as desirable frictions in learning as intentionally introduced, learner-controlled micro-interruptions that slow unreflective fluency at key moments (e.g., help-seeking, interpreting, or accepting AI outputs) to prompt metacognitive monitoring and evaluation. This concept of friction differs from desirable difficulties^132^, which primarily concern practice conditions that increase effort to improve long-term retention and transfer, and also, from productive failure^133^, which typically operates at the level of an instructional sequence that leverages pre-instruction problem solving and failure. By contrast, desirable frictions are interaction-level and light-touch design choices that can be embedded within successful performance to improve calibration, justification, and epistemic responsibility during AI-supported learning. For example, when learners seek support from AI, the desirable frictions in learning could be AI (now acting as scaffolding): (i) first checks whether it has correctly understood the learners’ intention before providing an answer; (ii) prompts learners to think about the criteria for evaluating the quality of the answer before and after receiving it; and (iii) remind learners to think about what judgments they made too easily during the previous interaction and might have been misled by the AI.

### Challenge the Pitfall of Pandering by Engineered Dissent

The **Pitfall of Pandering** refers to how GenAI is optimised to be agreeable and user-satisfying, but can also drift from supporting learners’ thinking to affirming learners’ beliefs. A central issue for this pitfall is that GenAI’s sycophancy may undermine learning^170^. GenAI may act as an echo chamber that mediates users’ stated views or preferred framings to gain approval^171^, producing a socially rewarding interaction that learners can misread as epistemic confirmation^170,172,173^. In educational settings, this is especially harmful because the goal is to refine learners’ judgment rather than merely produce agreeable feedback; yet pandering reduces productive disagreement, discourages misconception repair, and can strengthen false confidence. This risk is reinforced when learners treat the assistant as an epistemic authority and interpret affirmations as signs of understanding, thereby increasing trust and dependence in learning workflows^149,174^.

Within the SYNC model, we propose that pandering may accelerate metacognitive offloading by encouraging learners to outsource not only how to solve a problem, but also what to believe. When GenAI mirrors a learner’s stance, it can short-circuit orientation and evaluation, which may lead learners to stop asking what would support their claims, what alternative hypotheses exist, or which assumptions drive the conclusion^149^. The risk of such a pitfall compounds because GenAI can be highly persuasive in conversational settings, making agreeable messages particularly effective at shifting attitudes while preserving learners’ sense of personal control^175^. Meanwhile, learners may underestimate the extent to which AI shapes their judgments, creating a subtle feedback loop in which repeated interactions shift beliefs while maintaining a subjective sense of autonomy^176^. Recent evidence also suggests that GenAI can support customised or personalised content generation while, at the group scale, it can homogenise learners and reduce the diversity of creativity^33,44,170^, which is closely related to the pitfall of pandering.

In SYNC terms, escaping pandering requires reallocating roles so that AI support tools do not take over the learner’s metacognitive control. Instead of maximising agreement, effective AI support should introduce fresh insights beyond the echo chamber, and learning scaffolding should reinforce learners’ deliberate judgment and reflection. A SYNC-aligned approach is **engineered dissent** to convert the same conversational convenience that drives biased querying into structured practice that forces viewpoint expansion and verification^171^. In order to achieve engineered dissent, the AI support should (i) generate potential counterarguments based on data beyond what the learner provides, (ii) act as multiple AI agents that think or interact from different perspectives, and (iii) explicitly label AI’s assumption-dependence or uncertainty and rewording^149^; and the learning scaffolds should i) trigger learners’ pre-prompt commitments (e.g., what would change my mind and what is the quality criteria), (ii) suggest learners to monitor where the AI agreed, identify at least one disconfirming source, and revise after confronting counterevidence, and (iii) require learners to conduct AI-free reconstruction/transfer and reflect if there is over-alignment which often soften disagreement and omit counterpoints, reducing the cognitive friction that drives durable learning^171,174^.

### Beyond the Pitfall of Coherence by Balanced Aid

The **Pitfall of Coherence** refers to how GenAI’s outputs can appear internally consistent, well-structured, and narratively well-connected, thereby making unsupported or false claims feel plausible because they fit smoothly into a structured explanation. This coherence can also create a buzz-in effect because the answer already supplies a complete narrative, leaving learners with less need to decide what remains uncertain, what to ask next, or where the explanation may break down. Although convenient, such over-completion may trigger learners’ (meta)cognitive offloading, reducing their need to choose what to think next, what to ask, and what uncertainties to resolve. Thus, the risk of AI is no longer mainly hallucination alone but truthiness-by-coherence, where narrative consistency and confident extension substitute for evidence. Recent work on factuality stresses that LLMs can generate plausible but incorrect statements, and that coherent presentation can mask grounding failures and mislead users into over-trusting the content^88,173^. The harm is especially acute in learning where coherent errors are easy to internalise because they introduce misconceptions within what appears to be a reasonable and well-structured framework, which are harder to detect. Moreover, people often overestimate what LLMs know, thereby strengthening the coherence illusion and reducing verification^177^. This can be amplified in real-world interaction where users provide incomplete context, and LLMs still generate confident, coherent guidance, producing failure modes that benchmarks do not reveal^178^. When scaffolds are absent, learners may increasingly optimise for coherent products (e.g., well-written solutions) rather than epistemic correctness, which risks degrading durable learning even when short-term performance improves^92,149^.

The main difference between the pitfalls of coherence and fluency is that coherence here emphasises the often overly structured style of GenAI (e.g., bullet points, clean sections, polished conclusions), which can impose an external structure that narrows early exploration, reduces autonomy in inquiry, and closes orientation^31,179^, thereby encouraging metacognitive offloading. Even when AI is framed as scaffolding (rather than support), its overly structured guidance may become **over-scaffolding**, which over-directs planning, monitoring, and reflection and reduces metacognitive engagement. Over-scaffolding can also disrupt autonomy and weaken self-regulation, limiting or even degrading metacognitive development^124,180^. The goal is to provide necessary metacognitive scaffolding while preserving learners’ self-regulation^32^.

To achieve the **balanced aid**, AI support tools should default to the minimum necessary help (e.g., a single next-step hint, a diagnostic question, or an evidence check) and use progressive disclosure. In this way, deeper assistance is unlocked only after the learners actively request, monitor as necessary, or choose a direction, thereby preventing metacognitive offloading. For example, to avoid over-scaffolding, effective learning scaffolds in Quadrant II should structure the learning process just enough to keep learners engaged in just-in-time and just-enough metacognition, such as goal-setting, strategy selection, and reflection, while fading as competence grows to protect autonomy and self-regulation.

### Defuse the Pitfall of Transparency by Contextualised Verification

The **Pitfall of Transparency** refers to how GenAI’s visible rationales, step-by-step explanations, citations, or interface cues can make the system appear inspectable and accountable, leading learners to treat explanation visibility as a guarantee of correctness rather than as material for evaluation. In practice, transparency can become performative, which is a convincing justification that reduces scepticism and encourages acceptance. Research on over-trusting AI recommendations shows that users can become complacent (insufficiently monitoring or cross-checking) depending on how system features shape trust and perceived reliability^181^. Evidence from explainability research shows that even placebic (content-light) explanations can invoke trust similar to real explanations, implying that the presence of an explanation can function as a credibility cue rather than informative support^182^. More broadly, transparent explanations often increase acceptance regardless of correctness. In human-AI decision-making, explanations raised people’s tendency to follow the AI even when it was wrong, without producing performance gains^183^. With LLMs specifically, explanations increase reliance on both correct and incorrect responses, and can reduce users’ tendency to ask follow-up questions-”precisely the opposite of what transparency is intended to achieve^184^. In human learning, this can be harmful because such transparency may trigger learners’ cognitive and metacognitive offloading. For example, the transparent and explained answer may seem accountable even when it is incomplete, assumption-dependent, or wrong, weakening calibration and amplifying downstream errors^149,177^.

The SYNC model proposes that transparency may contribute to metacognitive offloading through an explanation-as-known heuristic whereby the felt availability of a rationale substitutes for checking. This effect is strengthened by authority-like framings of explanations (e.g., expert, logical, or empirical rationales), which can induce users to align with an AI judgment even when they cannot evaluate whether it is accurate^185^. This interacts with learners’ limited ability to evaluate LLMs’ competence. For example, when responses include visible rationales or explanatory cues, users may treat them as evidence of correctness, inflating confidence even without accuracy gains^177^. Therefore, when GenAI is embedded in the learning process as an authoritative role and provides seemingly transparent explanations, learners lacking cognitive foundations and metacognitive abilities will find it difficult not to offload their own judgment and monitoring to AI. Pedagogically, this can distort human-AI interaction because learners may lean on readily consumable AI explanations and guidance to move forward quickly rather than onload necessary metacognition (e.g., planning, monitoring), which can foster dependence and diminish durable learning^91,174^.

In SYNC terms, moving beyond transparency requires contextualised verification. Transparency should scaffold the learner’s metacognitive monitoring and evaluation rather than simply encourage trust or compliance. Research suggests that over-reliance is not simply an inevitable bias, but a strategic response to the benefits of verification^186^. To provide contextualized verification, AI support tools need to move beyond from acting as an authoritative, guiding, or even Socratic tutor, and instead act as an equal collaborator or negotiator, linking sources of information and addressing uncertainties within learner’s specific learning context (e.g., what is grounded vs inferred in certain context), while linking transparency and interpretability with verifiable actions (e.g., consider the distance between the source and the problem to be solved, compare two alternative mechanisms for specific learning task)^88,184^. Equally importantly, scaffolding should deliberately prompt learners to engage in metacognitive processes within specific learning contexts, rather than simply providing general features, principles, and strategies. For instance, Chen et al.^187^ found that feature-based explanations can reduce human-AI decision quality in AI-error cases by increasing reliance, whereas example-based explanations (more context specific) can better preserve independence and support complementary performance^187^. Therefore, balanced scaffolding should onload metacognition within contextualised cognition processes, such as requiring an initial independent attempt or structured checklists for specific cognition tasks, which can reduce autopilot performance compared to simple explainable AI^188^.

Although the four pitfalls may interact and can converge on similar outcomes, such as reduced verification and greater metacognitive offloading, they are distinguished by their proximal mechanisms. Fluency operates primarily through processing ease, whereby effortless interaction may inflate confidence; pandering operates through interpersonal agreement and affirmation, which may reduce critical disagreement; coherence operates through narrative and structural completeness, which may prematurely close further inquiry; and transparency operates through explanation-based credibility, whereby visible rationales or citations may be treated as evidence that an answer is already justified. Thus, the four pitfalls are not mutually exclusive, but describe different pathways through which AI may weaken learners’ metacognitive monitoring and control.

## Limitations and Conclusions

This perspective paper has several limitations. Most importantly, the SYNC model should be understood as a conceptual, theory-building framework rather than a prescriptive, empirically validated model. Although it draws on existing theories and emerging evidence to explain how learners may coordinate cognitive and metacognitive processes with AI support, its proposed mechanisms and outcomes have not yet been systematically tested. Future research is needed to operationalise the model, develop more testable hypotheses, and examine whether the proposed processes of human-AI synergy can be observed in different learning contexts.

Another related limitation concerns measurement. At present, few established measures or coding schemes exist for key constructs in the SYNC model, particularly metacognitive offloading and metacognitive onloading. While learners may delegate planning, monitoring, evaluation, or reflection to AI, it remains unclear how these processes can be reliably captured and distinguished from productive self-regulation or unproductive over-reliance. Future studies should therefore develop behavioural, self-report, trace-based, dialogue-based and performance-based indicators to assess when AI use supports, replaces, or strengthens learners’ metacognitive regulation.

The proposed SYNC model, metacognitive concepts and relevant hypotheses also require further examination across domains, learning contexts, task types and learner differences (e.g., prior knowledge, age, self-regulation, and AI literacy). Our discussion has focused mainly on contexts in which learners interact directly with AI, while paying less attention to classroom implementation, collaborative learning and teacher-mediated use. Also, human-AI synergy may differ across domains such as writing, mathematics, science, language learning, and programming, as well as across tasks such as problem solving, feedback interpretation, reflection, and creative production. These differences may influence what learners offload to AI and what they internalise through continued interaction.

In addition, our discussion centres on human learning and performance in human-AI interaction, rather than on AI’s performance itself, including how AI capabilities may evolve, whether AI can meaningfully demonstrate forms of metacognition, and how humans and AI might co-evolve toward human-AI synergy. Finally, the evidence base remains emerging, limiting strong claims about generalisability across educational, professional, and cultural contexts, especially given the rapid development of AI technologies.

In summary, this perspective defines concepts of metacognitive offloading and onloading, introduces the SYNC model to explain how GenAI can decouple task performance from durable learning, and identifies four pitfalls and approaches that may shift learners from autopilot performance to synergetic mastery. We propose that researchers, educators, and developers should co-design AI agents and learning environments with contingent, fading scaffolds and process-sensitive evaluations so that AI boosts productivity without eroding learning.

## Supplementary information

Transparent Peer Review file

## Data Availability

This article is a Perspective and does not report original research data. No datasets were generated or analysed.

## Footnotes

1 Both the Metacognitive Offloading and Metacognitive Onloading constructs are theoretically defined in this Perspective; established approaches to their measurement remain limited.
