# Supplementary material for: A metacognitive approach to learning and performance in human-AI interaction
Source: Commun Psychol. 2026 Oct 30;4:150. doi: 10.1038/s44271-026-00548-y (PMC13722690; doi:10.1038/s44271-026-00548-y)
Supplement: Supplementary file 1 [file 44271_2026_548_MOESM1_ESM.pdf]

nature portfolio

Peer Review File

A Metacognitive Approach to Learning and Performance in Human-AI Interaction

Corresponding Author: Professor Dragan Gasevic

This file contains all reviewer reports in order by version, followed by all author rebuttals in order by version.

Version 0:

Reviewer comments:

Reviewer #1

(Remarks to the Author)
The paper is very interesting to read and very promising! The possible separation between AI-supported task performance and durable human learning is very convincing. I find the distinction between cognitive offloading, metacognitive offloading, and metacognitive onloading promising, and the SYNC model offers a useful starting point for thinking about human-AI interaction in learning contexts. However, I think the manuscript requires substantial revision to make its contribution clearer, more focused, and conceptually sharper. Below, I provide my comments and suggestions for improvement section by section:

Introduction

• The introduction is well written, but it is too broad at the beginning. The historical framing could be shortened substantially.
• The main problem should appear earlier and more directly: AI may improve task performance without supporting durable learning.
• Please clarify already in the introduction what the unique contribution of the paper is.
• The relationship to existing frameworks should be stated more sharply: What does SYNC add beyond cognitive offloading, self-regulated learning, hybrid intelligence, and HHAIR - Hybrid Human–AI Regulation?

Section 2: Metacognitive Offloading and Onloading

• Very interesting new differentiation -> this is one of the strongest parts of the manuscript, the distinction between cognitive offloading and metacognitive offloading is useful and relevant.
• However, the conceptual boundary could be clearer. Please clarify when cognitive offloading becomes problematic metacognitive offloading.
• The term “metacognitive onloading” is promising, but it needs a more precise definition -> the term is missing in Box 1. It would help to provide observable examples of offloading and onloading in real learning situations.

Section 3: The SYNC Model

• The SYNC model is intuitive and potentially useful. The distinction between performance and learning is convincing.
• At times, the vertical axis appears to represent durable learning as an outcome, but it is also described in terms of scaffolding and metacognitive onloading. Similarly, the horizontal axis appears to represent performance, but it is also described in terms of AI support use and offloading. These are related but not identical constructs. Clarifying whether the axes refer to outcomes, instructional conditions, or learner processes would strengthen the conceptual precision of the model.
• Please explain more precisely what counts as “AI support” and what counts as “scaffolding”. The same AI system may sometimes provide support and sometimes scaffolding; this ambiguity should be addressed.
• The four quadrants are useful, but the descriptions are too long and partly repetitive. I suggest shortening the quadrant descriptions and summarising them in one clear table (see my next comments)
• Is the model mainly descriptive, explanatory, or prescriptive? If the model is prescriptive as well, the authors need to be more concrete about design principles and also more cautious: the paper should not imply that Quadrant IV is always best in every context without enough empirical evidence. If the model is explanatory, the authors need stronger theoretical precision and clearer causal assumptions (e.g. specify mechanisms, possible indicators)

Quadrant I: Floundering

• The idea is clear, but the section could be shortened.
• The main point is that low AI support and low scaffolding do not automatically produce productive struggle.
• Please distinguish more clearly between productive difficulty and unproductive floundering.
• One concrete example would be enough.

Quadrant II: Effortful Learning
• This section is convincing, but it overlaps with the general discussion of scaffolding.
• Please reduce repetition.
• The section should focus more clearly on why learning can be high even when AI support is low.
• It would help to explain when this quadrant is desirable and when it may be inefficient.
Quadrant III: Autopilot Performance
• This is probably the most important quadrant for the paper, very central
• The concept of "autopilot performance" is strong and should be highlighted more clearly and try to clarify how autopilot performance can be empirically identified. For example: high-quality output, low metacognitive engagement, weak transfer, poor AI-free performance.
Quadrant IV: Synergetic Mastery
• This quadrant is the normative goal of the paper.
• Please make more concrete what "synergetic mastery" looks like in practice.
• Try to add one or two worked examples, such as writing, programming, clinical reasoning, or scientific inquiry. Right now, the description is rather abstract.
• Please clarify how scaffolding is faded over time.
• See my comment before, is the model prescriptive? The authors should avoid making the quadrant sound idealised unless empirical evidence is stronger.
Section 4: Autopilot Performance Pitfalls
• The four pitfalls are interesting and useful. However, they partly overlap in my opinion: Fluency and coherence are especially close and should be more clearly distinguished. Transparency also overlaps with coherence when explanations appear well structured but are not valid.
• I suggest presenting the four pitfalls in a table with core mechanism; learning risk; example; design response. -> in particular, specific examples (how learners can avoid the risks might be quite useful)
• The text could then be shortened considerably.
Limitations and Conclusion
• The limitations should be more substantial.
• Please state more clearly that SYNC is a conceptual model, not yet an empirically validated model (emphasize it more)
• The lack of measures for metacognitive offloading/onloading should be discussed.
• Domain differences should be considered. Learner differences should also be considered, especially prior knowledge, age, self-regulation, and AI literacy.
• The conclusion should be shorter and more focused on the main contribution.

Reviewer #2

(Remarks to the Author)
This article presents a perspective on human-AI interaction. The key claim is that generative-AI overreliance can decouple task performance from skill learning, with the possibility that high performance can be accompanied by low learning. The authors conceptualise this in a model with separate dimensions for performance and learning. They highlight the danger of 'autopilot performance' (high performance and low learning) and suggest some design features that could lead to 'synergistic mastery' (high performance and high learning). The authors argue that this could be achieved via 'metacognitive onboarding'.

Overall, I think this is clearly an important topic that is of high interest to scientists working in the field (as well as the general public). Many of the points made by the authors seem plausible, but I was not fully convinced of the theoretical advance made in this work, or the suitability for Communications Psychology (at least in its present form).

1. The work comes across as somewhat speculative and imprecise. As the authors acknowledge, "we do not yet provide measurable outcomes or clear indicators for assessing constructs such as metacognitive offloading and onloading". The worry here is not so much about the model's empirical support, but more about originality and novelty of this work. It would be more persuasive if the authors could clearly explain how their model develops existing ideas about deskilling from gen-AI, and a good way to do this would be to present some clear diagnostic empirical predictions that would distinguish their approach from others that have already been presented. I found this somewhat lacking.

2. Another area where the novelty of this work could be further articulated would be if the authors could discuss whether their model applies only to gen-AI, or if the same would apply to other forms of cognitive assistance, like a student working with or without their textbook. If the model is specific to gen-AI, what makes gen-AI special?

3. The article contains many normative assertions, such as "we must emphasise learning environments where metacognition is intentionally onloaded through instructional design" (pp. 2-3) or "performance enhancement should not come at the expense of learning" (p. 17). There are not really backed up but they seem debatable. Must performance enhancement always bring learning too? There seem to be many uses of gen-AI where performance enhancement alone is enough (e.g. consider someone automating a boring work task that they have no intrinsic interest in learning to perform by themself, or a person using gen-AI to write the code to set up a simple website but with no desire to learn code on their own). To take another example, the authors say that it is problematic that "when a task is perceived as overly difficult or low-value, learners may fail to activate their metacognition" (p. 8). But why should they activate their metacognition for a task that has low-value, potentially requiring them to withdraw mental effort from a concurrent higher-value task? This, along with the pejorative phrase 'metacognitive laziness' gives the article a somewhat moralistic tone that I think could be explored further by discussing the question of when learning is desirable and when it is irrelevant, and whether the authors' SYNC model

applies to the latter or only the former. This would clarify the model's areas of applicability.

4. I did not find the review fully balanced. The authors highlight situations where use of external tools may cause confidence to drift upwards and unaided performance to drift down. But examples of the opposite patterns can be found too, for example Ferguson, McLeam & Risko (2015), who found that internet access can reduce feeling-of-knowing, and Lira, Rogers, Goldstein, Ungar, & Duckworth (2025), who found that gen-AI assistance led to improved performance on writing task even when the AI tool was removed.

5. I found the authors' depiction of their model in Figure 2 hard to understand. In particular, the top part of the top panel seems to suggest that 'high learning gain' and 'high task performance' should co-occur in a situation where 'learners cannot do' the task. I don't think that the authors mean to imply that high task performance and learning occur when someone cannot do a task, so I suggest removing the 'Learners cannot do' label.

Version 1:

Reviewer comments:

Reviewer #1

(Remarks to the Author)
I really enjoyed to read your theoritical concpets - your paper has a strong central idea and could make a useful conceptual contribution. I propose the following improvements to clarify and sharpen the theories:
- You should explain more precisely how metacognitive offloading differs from metacognitive laziness, weak self-regulation, overreliance, automation bias or the delegation of epistemic agency. The same applies to the relationship between onloading, metacognitive scaffolding and internalisation.
- I like your SYNC Model, however, I think it could be improved. The current model links the learning axis closely to scaffolding and the performance axis to AI support. Scaffolding and AI use are design conditions; learning and performance are outcomes. This distinction should be reflected in the model and figures
- The quadrant descriptions currently include assumed causes such as prior knowledge, level of AI support and scaffolding. These factors may lead to a quadrant, but they do not define it. The labels Effortful Learning and Synergetic Mastery also contain process and value assumptions that go beyond the two axes.
- The pathway from Effortful Learning to Synergetic Mastery is plausible but remains hypothetical. Other trajectories may occur depending on prior knowledge, task type, system quality, motivation and the timing of support. Try to visualize this uncertainty in the figure
- Furthermore, it remains unclear whether a quadrant describes an interaction episode, a complete task, a learner or a longer developmental trajectory. Learners may move between quadrants within the same task.
- According to the pitfalls: At first, I really liked your typology. However, fluency, coherence and transparency overlap because each can increase trust and reduce verification. You could try to identify the distinct mechanism behind each pitfall and explain how the categories relate to one another.
- The figures of the four pitfalls are also dense, it takes some time to understand, try to simplify
- The role of scaffolding seems to be convincing, however, it is right unclear - it is
teacher-, peer- or AI-generated scaffolds? AI-generated support can itself be inaccurate, overly directive or poorly aligned. The framework should specify how scaffolds are validated, adapted and faded.
- Prior knowledge, domain, task stakes, verifiability, learner age and AI literacy currently appear across different sections. These factors could be presented systematically as moderators.
- The manuscript contains grammatical errors and unfinished reference information, including an editorial placeholder in the reference list -> Bjork RA, Bjork EL. Desirable difficulties in theory and practice. Journal of Applied Research in Memory and Cognition. 2020;9(4):475–479. "DOI varies by indexing source; add if you confirm from your preferred database.

Reviewer #2

(Remarks to the Author)
I'm grateful to the authors for this responsive revision, which I think has improved on the initial submission.

Open Access This Peer Review File is licensed under a Creative Commons Attribution 4.0 International License, which permits use, sharing, adaptation, distribution and reproduction in any medium or format, as long as you give appropriate credit to the original author(s) and the source, provide a link to the Creative Commons license, and indicate if changes were made.
In cases where reviewers are anonymous, credit should be given to 'Anonymous Referee' and the source.
The images or other third party material in this Peer Review File are included in the article's Creative Commons license, unless indicated otherwise in a credit line to the material. If material is not included in the article's Creative Commons license and your intended use is not permitted by statutory regulation or exceeds the permitted use, you will need to obtain permission directly from the copyright holder.
To view a copy of this license, visit https://creativecommons.org/licenses/by/4.0/
